# Quantifying the impacts of posture changes on office worker productivity: an exploratory study using effective computer interactions as a real-time indicator

**DOI:** 10.1186/s12889-023-17100-w

**Published:** 2023-11-08

**Authors:** Hong Wang, Diran Yu, Yu Zeng, Tongyu Zhou, Weixiang Wang, Xuan Liu, Zhichao Pei, Yumeng Yu, Chaoju Wang, Yingqi Deng, Ali Cheshmehzangi

**Affiliations:** 1 China Academy of Building Research (CABR), 30 North Third East Road, Beijing, 100013 China; 2 National Engineering Research Center of Building Technology, 30 North Third East Road, Beijing, 100013 China; 3 Buro Happold Asia, No. 39 East 3rd Ring Road, Beijing, 100022 China; 4 The Bartlett School of Sustainable Construction, University College London (UCL), London, WC1E 6BT UK; 5 Department of Architecture and Built Environment, University of Nottingham Ningbo China, 199 Taikang East Road, Ningbo, 315100 China; 6 College of Architecture, Qingdao City University (QCU), 79 Tieqishan Road, Qingdao, 266106 China; 7 Network for Education and Research on Peace and Sustainability (NERPS), Hiroshima University, A601-(3), 1-3-1 Kagamiyama, Higashi-Hiroshima, Hiroshima, 739-8530 Japan

**Keywords:** Sit-stand desk, Work productivity, Office workers, Quantitative approach, Computer interaction, Postures

## Abstract

**Background:**

Working in a standing posture is considered to improve musculoskeletal comfort and can help enhance office workers’ performance in the long term. However, there is a lack of a quantitative, real-time measure that reflects on whether office workers can immediately become more concentrated and work more efficiently when they switch to a standing posture.

**Methods:**

To tackle this problem, this study proposed that the number of effective computer interactions could be used as a real-time indicator to measure the productivity of office workers whose work is primarily computer-based. Using this metric, we conducted an exploratory study to investigate the correlation between posture and productivity changes at a 10-minute resolution for eight participants.

**Results:**

The study found that when allowed to use sit-stand desks to adjust postures, participants chose to switch to standing posture for about 47 min on average once a day; standing work was most frequent between 2:30 − 4:00 pm, followed by 10:30 − 11:30 am, during which time the number of computer interactions also became higher, showing a significant positive correlation. In addition, participants were approximately 6.5% more productive than when they could only work in a sitting posture.

**Conclusion:**

This study revealed that posture changes could have an immediate improvement in productivity.

## Background

A sedentary work style is common for people working in offices. The majority of office tasks are computer-based, and most people work in a sitting posture. In middle-income and high-income countries, office workers often sit still for half of the day [1], which makes them a risk group for sitting-related diseases. The prolonged use of desktop computers and insufficient physical activity are associated with not only musculoskeletal discomfort, but also other health threats such as metabolic, cardiovascular and mental disorders [2, 3], and all-cause mortality [4]. Furthermore, There is also some evidence that prolonged sitting at work is related to a reduced ability to work productively [5] and detrimental to work engagement [6].

In light of this, reducing the sedentary work style of office workers has become a priority, with a growing number of interventions aimed at minimising total sitting time and duration of sitting bouts. Office workers were encouraged to be more physically active through changes in their work postures [7]. Many studies [8–10] suggested that changing postures, i.e., from sitting to standing or walking, could increase physical activity and elevate energy expenditure.

A conventional sitting desk can be replaced with a height-adjustable sit-stand desk and allows its user to alternate posture between sitting and standing as needed. The provision of sit-stand desks at workplaces is thought to be a sustainable solution to the problem of prolonged sitting. A systematic literature view [10] has found that sit-stand desks can reduce daily sitting time by approximately one to two hours. With the prevalence of finding the effects of using sit-stand desks, many different perspectives have emerged. In addition to those health-related branches, such as physiological health and discomfort [11], work productivity has received more and more attention [12, 13].

Work productivity is an important outcome measure that can be used in assessing the effectiveness of changing work postures. Some intervention studies [6, 14] pointed out that using sit-stand desks has shown little effect on productivity, while others [15, 16] have reported positive effects. It has to be recognised that work productivity is a person’s overall performance, and it can be influenced by many aspects, such as one’s physical condition, mental condition, personality, experience, and so on. Most productivity measures were based either on subjective questionnaires or on task completion time. However, the former can be less reliable due to the mental fatigue and acquiescence effect of the respondents after they repeatedly answer the questionnaire [17]; while the latter lacks quantitative analysis at a more microscopic time scale, e.g., whether there is an impact on productivity when working in a standing or sitting posture during the day.

In order to investigate the immediate effect of postures on work productivity, some studies have attempted direct measurements of brain activity. For instance, Nicholas Gilson et al. [18] examined the electroencephalogram (EEG) signals of subjects under different work conditions. They observed that sit-stand conditions showed the strongest EEG signals, followed by sit-walk and sit-only conditions. Similar findings were reported by Ju-Yeon Jung et al. [19] that brain efficiency was higher in a standing posture than in a sitting posture and supine posture. Although the use of an EEG can facilitate quantitative analysis of productivity, wearing an EEG device will inevitably have an impact on the daily work of the test subject; and cannot be used for long-term monitoring.

From the literature reviewed, it’s clear that most current research approaches the relationship between postures and work productivity from a macroscopic, qualitative angle. Prolonged periods of sitting have been linked to various health issues, and interventions like sit-stand desks have demonstrated the potential to reduce daily sitting time, thereby possibly mitigating some health risks associated with prolonged sitting. However, the precise impact of posture changes on work productivity is still under discussion. Traditional productivity assessment methods, such as subjective questionnaires or task completion times, have inherent limitations. Subjective questionnaires, for instance, can introduce bias due to individual perceptions influenced by various factors, while task completion times might not always reflect the quality or complexity of work. Moreover, these methods can be inconsistent across different tasks and might not provide real-time data, leading to potential inaccuracies. Recently, there has been growing interest in more direct, quantitative measures like EEG signals. Preliminary studies suggest that changes in posture can influence brain activity, with findings indicating that sit-stand postures produce more stronger EEG signals than other postures, especially during tasks like the N-back working memory test [19]. Yet, the invasive nature of such methods poses challenges. These findings highlight the complex relationship between posture changes and productivity, emphasizing the need for more detailed, quantitative research methods. Currently, there is a gap in offering a quantitative, real-time reflection of the immediate impact of posture changes on work productivity.

To tackle this problem, a non-invasive approach is necessary for work productivity measurement. Given that computer use is the dominant work in offices, it is tempting to ask whether we can measure the number of effective interactions with computers per unit of time as a quantitative indicator of work productivity. The main ways in which people interact with computers are by using the keyboard, clicking the mouse and moving the mouse. We consider keystrokes and mouse clicks to be conscious decision-making behaviours [20, 21] and can be regarded as effective interactions. On the other hand, moving the mouse may be an unconscious action, not necessarily a decision, so it is not considered an effective interaction. The higher the number of effective interactions per unit of time, the more productive one is; conversely, the lower the number of interactions per unit of time, the less productive one is. It is important to note, however, that as different people have different working styles, and the complexity of the task also influences the number of effective interactions, the results have to be normalised when comparing the work efficiency of different people. With appropriate monitoring software, the number of keyboard and mouse uses can be logged, and a non-intrusive productivity measurement can be achieved.

In light of this, the aim of this work was to investigate the immediate effect of different postures on work productivity, and an exploratory study was carried out in a real office workplace. We examined the office workers’ productivity at standing and sitting postures by quantitatively measuring the effective computer interactions. To ensure that the data collection process did not interfere with daily work, this study was conducted in a non-invasive manner. This study offers new perspectives and methods for exploring the relationship between posture and productivity, and its findings can help to build a better working environment for office workers.

## Methods

### Rationale for research design

The research is exploratory, examining the immediate relationship between postures and work productivity. Due to the inherent individual differences in work capability, while comparisons among individuals are important, monitoring each individual’s changes when switching postures is even more crucial. To mitigate the influence of external factors on productivity, such as changes in the nature of work tasks, the study was conducted during a period when participants were engaged in a consistent work activity. Given the challenges associated with real-time monitoring and maintaining a homogeneous group, the study was designed as a pilot.

A group of eight individuals from an architectural design team was selected as research subjects. During the six-week experimental phase, all participants worked on a unified architectural design project in the same office environment. This arrangement ensured uniformity in work content, with all participants using CAD drawing software and participating in document writing.

The primary dependent variable in the study is work productivity, measured by the number of effective computer interactions every 10 min, as recorded by the in-house software. This interval was chosen to ensure a balance between accuracy and sensitivity. Independent variables, such as posture time ratio and work posture restrictions, were identified based on their potential influence on productivity. Control variables included the working environment, psychological work satisfaction, and BMI (Body Mass Index), assessed using a mix of metrics and questionnaires. Throughout the six-week period, 6,216 data sets were gathered, ensuring a rich data collection and analysis. Further details about the participants, work conditions, productivity metrics, and the in-house software are elaborated upon in the subsequent sections.

### Participants and work conditions

This study was conducted in June and July 2022, at the Living Lab for Healthy Building and Workplace Productivity of China Academy of Building Research, in collaboration with the University of Nottingham Ningbo China. Eligible participants were eight full-time employees of the living laboratory, all of whom were working on the same architectural design project using CAD drawing and Word software during the study. They were briefed on the purpose of the measurement, risks and disclosure of data before data collection began. Prior approval for the implementation of this study was obtained from the Research Ethics Review Board of the University of Nottingham Ningbo China. The authors declare that all experiments were carried out in accordance with the relevant guidelines and regulations. All participants gave informed consent to the study.

In this 6-week test, the eight participants were divided into two groups, as shown in Table 1. The participants labelled A, B, C and D were in the control group and allowed to switch postures as needed throughout the whole test; the other 4 participants, namely E, F, G, and H, were in the intervention group that was requested to use only a sitting posture for the first three weeks and then to switch postures freely for the next three weeks. Before the test, all the participants had used sit-stand desks for more than half a year in the office, indicating they were already familiar with the use of such desks [22]. The initiation of a sitting-only phase for the intervention group was shaped by the methodologies of Baker et al., who began their study with a period of seated work [23]. To harmonize any pre-existing work habits among participants, this phase was extended to three weeks in the current study. This methodological approach was intended to ensure that the subsequent phase, which allowed for free posture switching, would produce an unbiased representation of the relationship between posture alterations and productivity.

**Table 1:** Restrictions on work postures

Participants	Restrictions on work postures	
	**Test week 1 ~ 3**	**Test week 4 ~ 6**
A, B, C and D	No limit	No limit
E, F, G and H	In sitting posture only	No limit

### Use of sit-stand desks

**Fig. 1:**
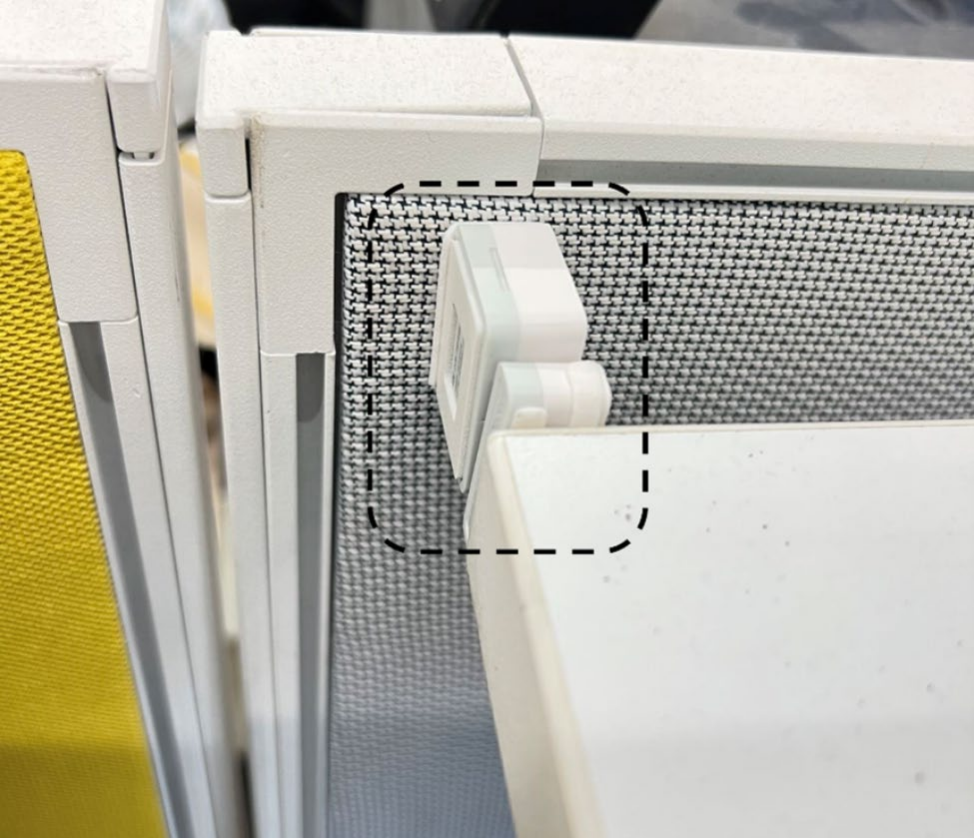
Xiaomi displacement sensor mounted on a sit-stand desk

In this study, the participants changed their work postures by using sit-stand desks. The term *use of sit-stand desks* refers to changing from a sitting posture to a standing posture. Eight Steelcase electric height-adjustable desks (Sit2Stand) were used in the study, with an adjustable height range from 705 to 1155 mm. The frequency of using sit-stand desks was recorded to determine whether the participants were working in a standing or sitting posture. Xiaomi displacement sensors (as shown in Fig. 1) were mounted on each participant’s sit-stand desk to automatically detect and record the status of the desks.

### Research design

In order to reproduce the real working conditions, this test did not place any restrictions on the working conditions of the participants. During the test, some participants were away from the office on business trips, etc., so the number of days of participation varied for each participant. In data analysis, individual results were normalised on a daily basis before they were compared with others.

Both qualitative and quantitative approaches were used in this study. The qualitative analysis was carried out utilising a questionnaire survey, which was used to assess the work satisfaction of the participants during the six weeks. The body mass index (BMI) changes were also recorded. In addition, a set of questions assessed participants’ musculoskeletal sensations, using a scale of discomfort, moderate, and comfort, marked as -1, 0, and 1, respectively. The quantitative approach focused on monitoring the number of computer interactions during the daily working hours (9 am-12 am and 2 pm-6 pm), and the use of sit-stand desks during these hours.

#### Psychological questionnaire and BMI records

Significant changes in psychological and physical conditions can have a great impact on individuals’ work productivity. Therefore, in this study, the work satisfaction and BMI changes of all eight participants were assessed to determine whether they were affected by these two factors.

The assessment of work satisfaction was made through a questionnaire consisting of four questions on work satisfaction, each measured using a Likert scale (1 to 5), as shown in Table 2. The participants filled out the form twice a week during the test. At the same time, they also registered their heights and weights at the time, which were then used to calculate their BMI index.

**Table 2:** Questions on work satisfaction

Questions on work satisfaction	Very disagree – very agree (1 ~ 5)				
I am satisfied with the workplace condition	1	2	3	4	5
I feel energised throughout the day	1	2	3	4	5
I feel positive at work	1	2	3	4	5
I have not encountered major obstacles in my work	1	2	3	4	5

#### Musculoskeletal comfort

When a person’s musculoskeletal is in a comfortable state, it positively affects work productivity [5]. Typical musculoskeletal discomforts for office workers are related to regions of the neck, back, knee, and ankle [11]. In order to investigate whether switching postures helps to improve musculoskeletal conditions, the intervention group was asked to describe their musculoskeletal comfort twice a week, using discomfort, moderate, and comfort (marked as -1, 0, and 1, respectively). Before the main study, a pilot test was conducted with a separate group of individuals to ensure the clarity and relevance of the questions.

An internal consistency analysis was conducted to ensure the reliability of the questionnaire used to measure changes in the musculoskeletal sensation of the participants. Internal consistency reflects the extent to which items of a test measure various aspects of the same characteristic and yield consistent results. Cronbach’s alpha coefficient [24], a widely recognized statistical measure, was employed to assess the internal consistency of our questionnaire. An alpha coefficient above 0.7 is generally considered an acceptable threshold for reliability.

After administering the questionnaire to the participants, the responses were analysed, and a Cronbach’s alpha value of 0.835 was obtained. This indicates that the questionnaire exhibits good internal consistency and is a reliable tool for capturing the musculoskeletal sensations of the participants in this study context.

#### Effective computer interactions

To study work productivity, we developed in-house software that records the number of effective computer interactions from participants over a 7-hour working period. In the context of computer usage, an “effective interaction” refers to specific actions that signify a user’s intent to communicate or give a command to the computer [25]. These actions include pressing a key on the keyboard, representing data input or a command such as typing text, using shortcuts, or executing specific functions, and clicking the mouse, which indicates a direct command, such as selecting an item, opening a file, or initiating a program. The frequency of these interactions serves as a proxy for productivity in tasks that primarily involve computer use. Conversely, certain actions, such as moving or scrolling the mouse, are not categorized as “effective interactions”. These are often passive actions, indicating the user is browsing or navigating the screen or trying to locate something within a page or document.

The software auto-starts upon system boot and performs daily data backups to ensure data reliability. For privacy concerns, the software only records whether an effective interaction takes place, without reading the exact keyboard input. The minimum recording time is 1 s, and a Boolean feature (0, 1) is used to indicate if an effective interaction occurs. To ensure the accuracy of our productivity measurements, our software is designed to pause recording when no keyboard or mouse activity is detected for a continuous period of 3 minutes. This feature was integrated to exclude potential non-work-related periods, such as coffee or toilet breaks. By implementing this 3-minute idle time cutoff, we aimed to ensure that our productivity data is solely based on active work periods, thereby enhancing the reliability of our findings.

#### Work productivity indicator

The main indicator of work productivity in this study is the number of effective interactions per 10-minute intervals. This interval allowed us to maintain optimal accuracy and sensitivity in measuring variations in productivity. If an effective interaction occurs more than once within a one-second period, the Boolean feature’s value is set to 1; otherwise, it is 0. Over the 7-hour working period, the value of effective daily interaction can vary between 0 and 25,200.

To account for natural differences among individuals, interaction values were normalized between 0 and 1. The ratio of the current number of interactions at any 10-minute time slot, *I*_*current*_, to the maximum number of interactions, *I*_*max*_, is used to describe the current work productivity of a person, as shown in the equation below:

1 $${P}_{x}=\frac{{I}_{current, x}}{{I}_{max, x}}$$

### Statistical analysis

Controlled variables, such as work satisfaction and BMI of each participant, were first subjected to descriptive statistics to determine their tendencies. This was done to ensure that there were no significant changes in psychological and physical conditions that could impact work productivity over the experimental period.

Work productivity data were normalized between 0 and 1 to account for inherent differences among individuals. Correlation analyses were employed to assess the relationships between work productivity and posture. A significance level (alpha) of 0.05 was set, with any p-value less than 0.05 being considered statistically significant. All statistical analyses were conducted using the data analysis tool, Origin 2021.

## Results and discussion

### Work satisfaction and BMI changes

As can be seen from the statistics in Table 3 below, there was no significant change in work mood for all participants over the six weeks of testing. The average value of each varied due to natural differences between individuals, but the coefficients of variation were all in small ranges. It can therefore be assumed that none of the participants was subjected to dramatic psychological or emotional changes during the test that could affect their work productivity.

Similarly, the BMI records reflected the absence of drastic physical changes in all participants during the six weeks, thus also ruling out any impact on productivity due to physical changes.

**Table 3:** Work satisfaction and BMI

Participants	Overall work satisfaction		BMI	
	Mean	Coefficient of variation	Mean	Coefficient of variation
A	4.46 (95%CI = 4.29–4.62)	0.058	16.87 (95%CI = 16.79–16.94)	0.007
B	4.00 (95%CI = 4.00–4.00)	0.000	27.30 (95%CI = 27.16–27.45)	0.008
C	4.83 (95%CI = 4.68–4.99)	0.051	22.14 (95%CI = 22.05–22.22)	0.006
D	4.38 (95%CI = 4.08–4.67)	0.081	21.86 (95%CI = 21.56–22.15)	0.016
E	4.39 (95%CI = 4.16–4.62)	0.078	19.85 (95%CI = 19.77–19.94)	0.006
F	3.62 (95%CI = 3.35–3.88)	0.110	22.24 (95%CI = 22.09–22.38)	0.010
G	3.27 (95%CI = 2.99–3.55)	0.130	23.76 (95%CI = 23.59–23.93)	0.011
H	3.83 (95%CI = 3.64–4.01)	0.070	25.64 (95%CI = 25.51–25.77)	0.007

### Musculoskeletal comfort

The results revealed a general improvement in musculoskeletal discomfort after the use of sit-stand desks, as shown in Table 4. The most significant improvement was in the relief of back pain, with all participants reporting increased comfort in their back muscles in the second three weeks when they were able to use the sit-desks freely compared to the first three weeks when they could only work in a sitting posture. This was followed by relief of neck pain, where 3/4 of the participants felt that there was an improvement. The last one, knee and ankle, varied among participants, with some perceiving an improvement, some perceiving little change, and some perceiving deterioration.

**Table 4:** Improvement in musculoskeletal comfort

Participants	Neck		Back		Knee and ankle		Overall	
	week 1 ~ 3	week 4 ~ 6	week 1 ~ 3	week 4 ~ 6	week 1 ~ 3	week 4 ~ 6	week 1 ~ 3	week 4 ~ 6
E	-0.167	0^↑^	-1	0.2^↑^	0	-0.2	-0.389	0^↑^
F	-0.5	-0.6	-0.833	0.6^↑^	0	0.4^↑^	-0.444	0.133^↑^
G	-0.333	0^↑^	-0.167	0.4^↑^	0	0	-0.167	0.133^↑^
H	-0.333	0^↑^	-0.167	0^↑^	0	-0.25	-0.167	-0.083^↑^

Legend: ^↑^ indicates an improvement

### Preference for sitting and standing posture

It was up to individual preference when to adjust posture throughout the day, and it was reflected by recording the use of sit-stand desks. As can be seen from Table 5, Participant B used sit stand desk on average 0.32 times a day, i.e., once every three days, while participant F used it more often with an average of 1.31 times a day. The average number of using sit-stand desks per day is 0.92, which is close to one use per day. And the length of each use ranged from about 35 to 75 min, with an average of about 47 min. The ratio of time spent standing and sitting is approximately 1:11. Sitting is still the primary working posture.

**Table 5:** The usage of sit-stand desks (SSD)

Participants	Number of days in the office	Number of times using SSD	Using SSD per day	Average length per use (mins/use)	Standing work per day (mins)	Ratio of standing against sitting
A	28	32	1.14	52.59	60.11	1:6.0
B	28	9	0.32	73.78	23.71	1:16.7
C	26	39	1.50	36.49	54.73	1:6.6
D	15	11	0.73	72.09	52.87	1:6.9
E	14	10	0.71	33.5	23.93	1:16.6
F	13	17	1.31	33.88	44.31	1:8.4
G	12	13	1.08	34.92	37.83	1:9.7
H	12	7	0.58	41.14	24.00	1:16.6
Average	18.5	17.25	0.92	47.30	40.19	1:10.9

The Spearman correlation coefficient for the average number of uses per day and the average length of time per use is -0.381, but the p-value is 0.352, greater than 0.05. Therefore, it cannot be concluded that the fewer times a person uses a sit-stand desk, the longer the single use would be. On the other hand, the Spearman coefficient for the average number of uses per day and the average length of use per day was 0.762, with a p-value of 0.028, showing a significant positive correlation. Combining the two points above, we can conclude that:

- The duration of working in a standing posture (i.e. time length per use of sit-stand desk) was not necessarily related to the frequency of using the sit-stand desk. Overall, the average single standing time for all participants was approximately 47 min;
- The more frequently one used a sit-stand desk, the longer one’s total daily standing time tended to be.

**Fig. 2:**
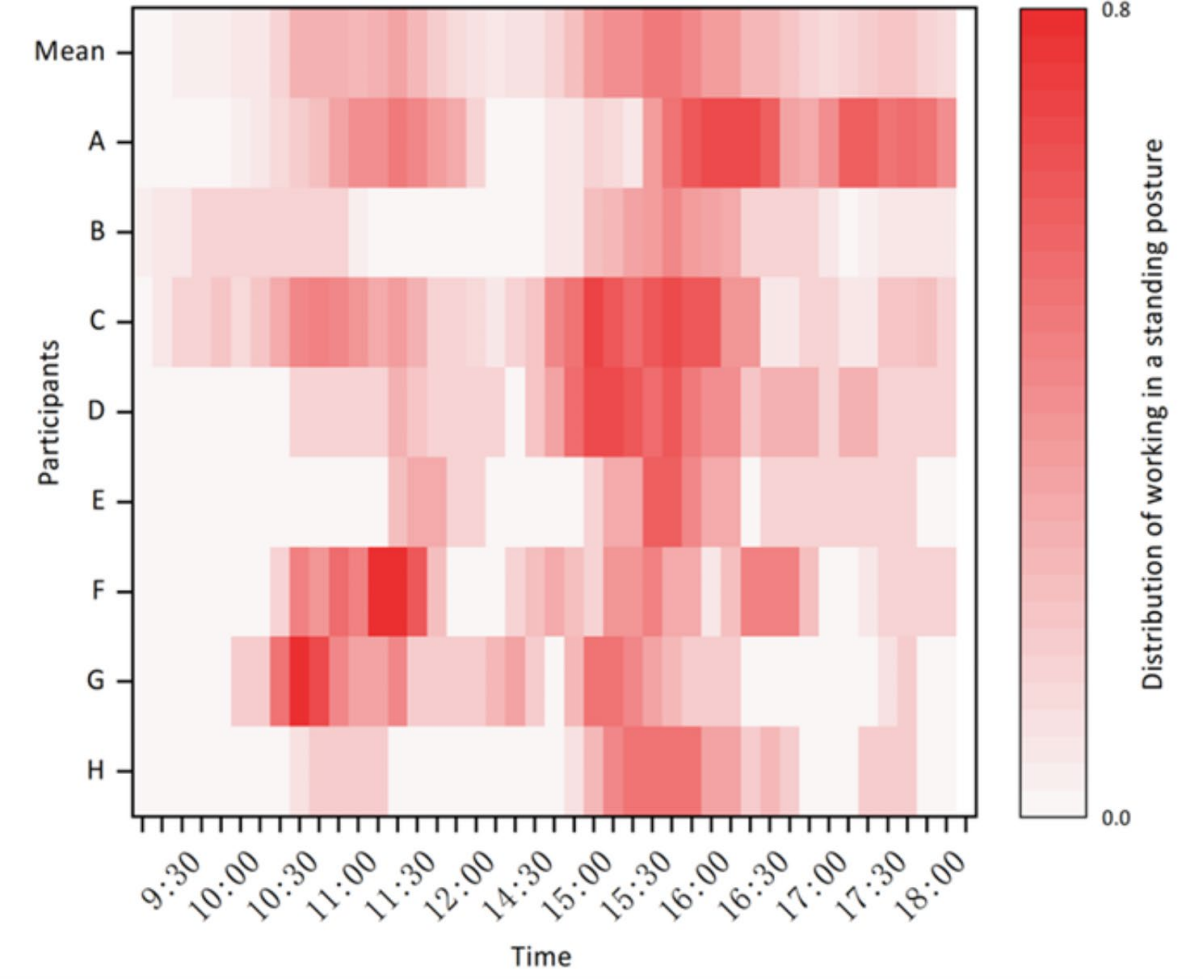
The time and frequency distribution of standing and sitting postures of all participants

Figure 2 shows the time and frequency distribution of standing and sitting postures of all participants when allowed to freely use the sit-stand desks. This statistic includes instances where the sit-stand desk was not used during the working day and reflects the willingness of participants to use the sit-stand desk. The data has been normalised on a daily basis to reflect the average use of sit-stand desks by participants during their respective participation in the test. The deeper the colour, the more frequently the participants used the sit-stand desks during the test; the lighter the colour, the less frequently ones used it. If the frequency is 1, then the participant worked in a standing posture at that time every day in the test, and conversely, if the frequency is 0, then the participants did not use the sit-stand desk (i.e. remained in a sitting posture) during that time period on all test days. As can be seen in Fig. 2, there is a clear pattern in the average use of the sit-stand desk, mainly between the hours of 10:30 − 11:30 am and 2:30 − 4:00 pm.

### The relationship between standing/sitting posture and productivity in a day

**Fig. 3:**
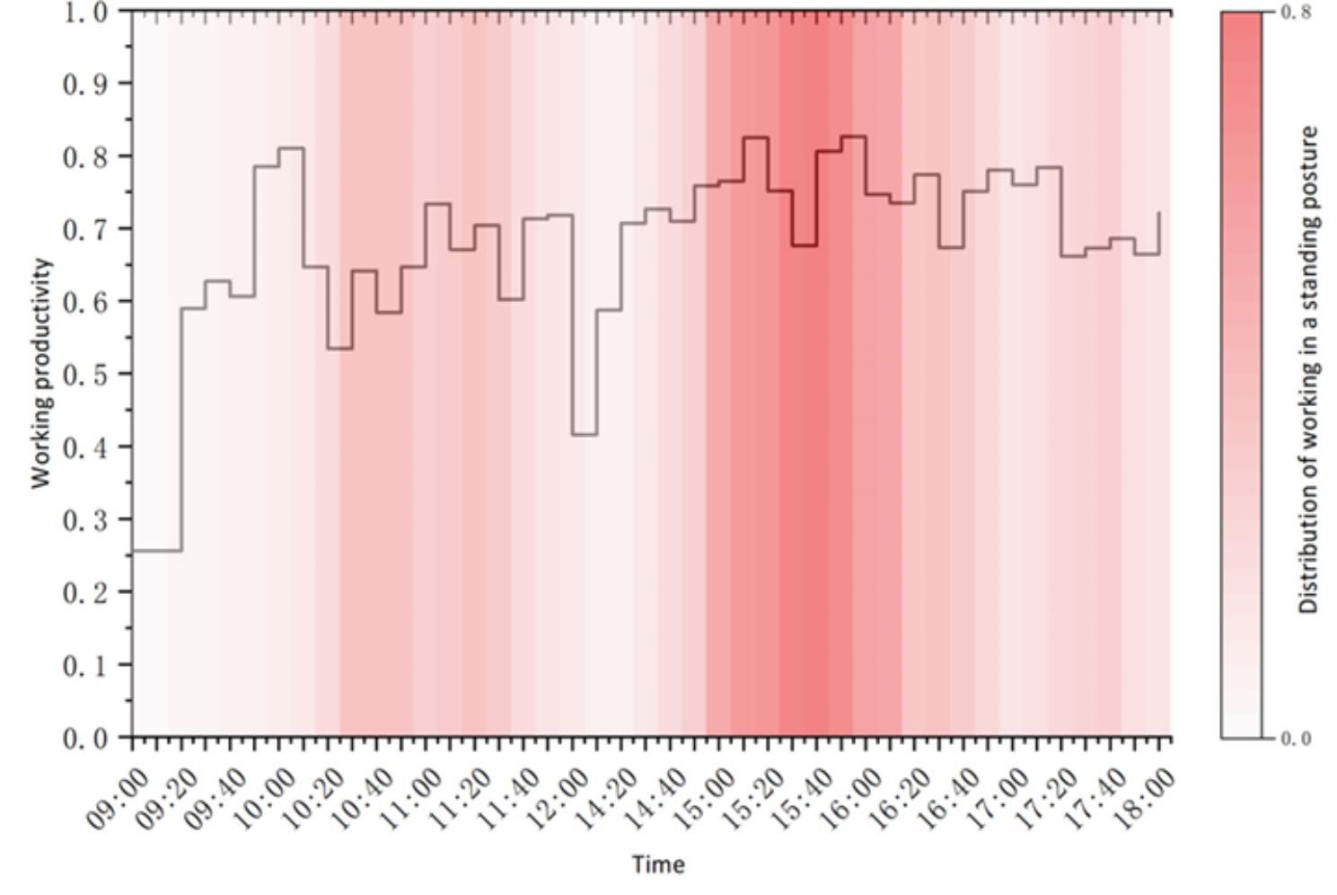
The average work productivity against the distribution of work in a standing posture (all participants)

In our analysis, we examined the relationship between two primary variables: the average productivity of the eight participants and the distribution of their standing work. When overlaying these two variables, as depicted in Fig. 3, a clear correlation emerges. The Pearson correlation coefficient, which measures the strength and direction of the linear relationship between these two variables is 0.436, with a significance p-value (0.004) much less than the standard threshold of 0.05. This suggests a moderate positive correlation between the two. At 9 am, when the participants just began to work, their average productivity was at its lowest point of the day, then showed a significant increase and reached its first peak of the day at around 10 am before starting to decline. This can be explained by the fact that at the beginning of the day, everyone was very energetic, and therefore, quickly went into a state of high productivity. Still, after an hour, the first peak was over, and productivity gradually decreased. At this point, many participants began to choose to work in a standing posture, and their productivity again increased. After 11:30 am, productivity quickly dropped to its second lowest point of the day as people’s attention began to drift away from work as they approached their lunch break. After a sufficient lunch break, the participants resumed work at 2 pm, and their productivity increased once again. From 2:30 pm onwards, the peak probability of work in the standing posture was reached, and, at the same time, the productivity of the day was simultaneously at its highest. When it got to 4 pm and beyond, productivity decreased as everyone gradually returned to work in a sitting posture. It was clear at the 10-minute resolution that there was a noticeable increase in the productivity after the participants adopted the standing posture.

### The impact of postures on work productivity

**Fig. 4:**
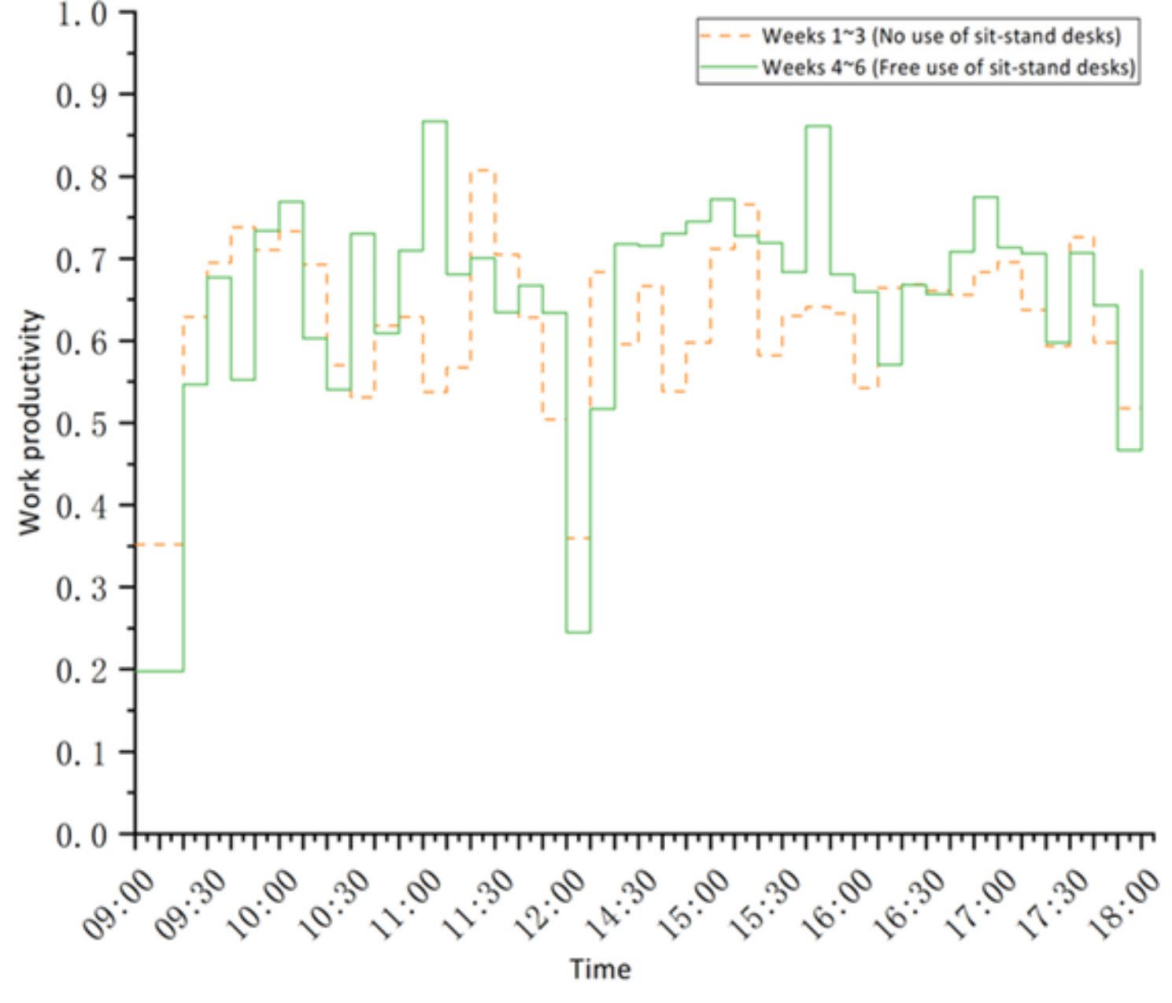
The average daily work productivity with and without using sit-stand desks (intervention group)

**Fig. 5:**
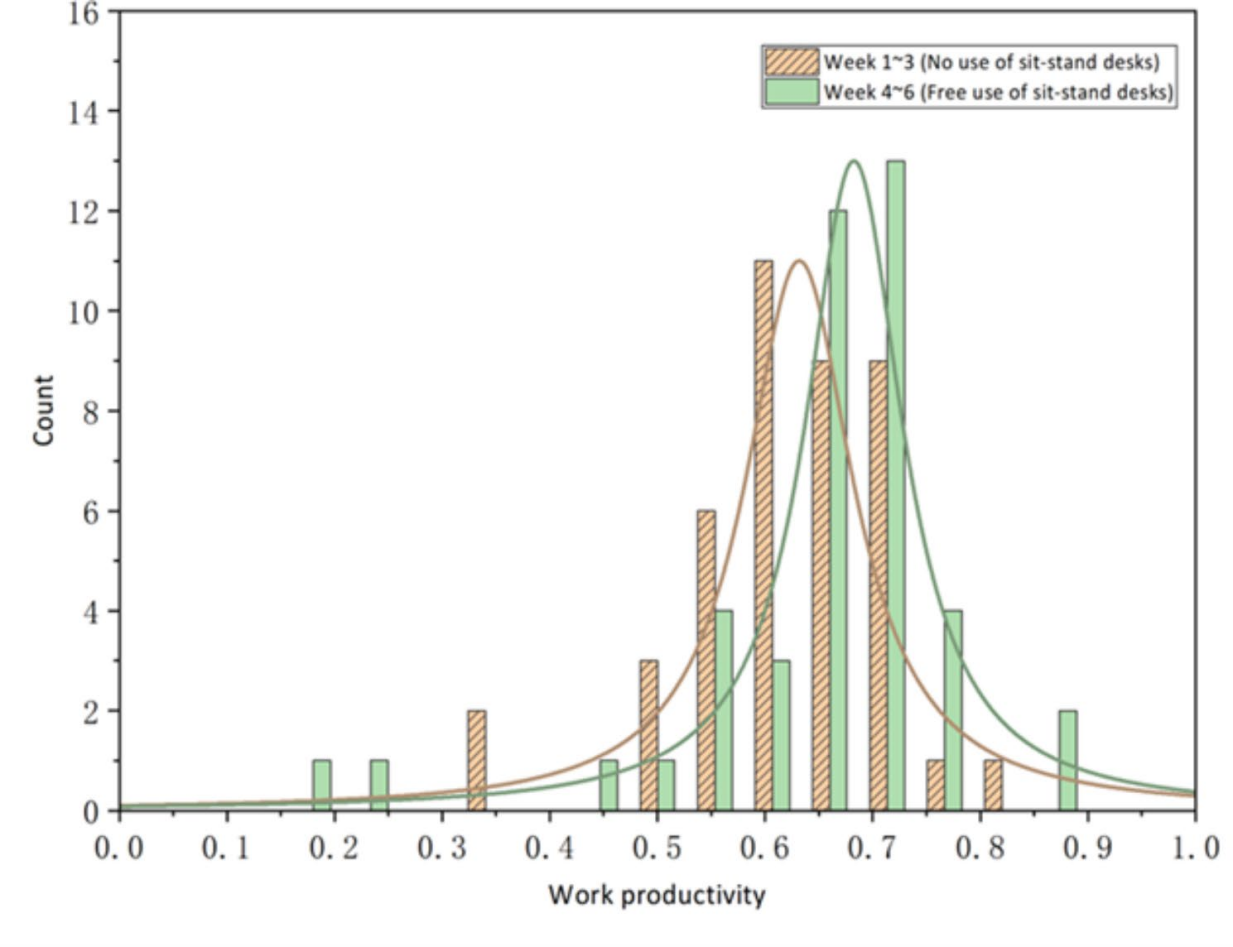
The distribution of work productivity per ten minutes with and without using sit-stand desks (intervention group)

Comparing the performance of the intervention group, this study found that the use of sit-stand desks could help promote overall productivity as shown in Table 6. In weeks 1 to 3, when the intervention group was requested to work in a sitting posture, the mean work productivity was 0.62 (95%CI = 0.59–0.65), while in weeks 4 to 6 when they were allowed to use sit-stand desks freely as needed, the mean work productivity was 0.66 (95%CI = 0.61–0.69), with an improvement of approximately 6.5%. Figure 4 depicts the mean work productivity between weeks 1 to 3 and weeks 4 to 6. It can be seen that in the two time periods (i.e., 10:30 − 11:30 am and 2:30 − 4:00 pm) when sit-stand desks were used more frequently, the participants’ work productivity was also higher, while in the other time periods the productivity difference was much smaller. It can be explained that when working in a standing posture, people tend to be more concentrated and hence the higher productivity than working in a sitting posture. The effect of using sit-stand desks on increasing productivity can also be seen in Fig. 5. It displays the distribution of the number of occurrences of different levels of productivity per ten minutes. When the restriction on posture was removed and participants had free access to their sit-stand desks, there was a significant increase in the number of occurrences of high productivity.

**Table 6:** Comparison of work productivity during different time intervals across weeks 1–3 and weeks 4–6

Time	Weeks 1 ~ 3	Weeks 4 ~ 6	Time	Weeks 1 ~ 3	Weeks 4 ~ 6
	Work productivity			Work productivity	
9:00–9:10	0.35	0.20	14:00–14:10	0.68	0.52
9:10 − 9:20	0.63	0.55	14:10–14:20	0.60	0.72
9:20 − 9:30	0.70	0.68	14:20 − 14:30	0.67	0.72
9:30 − 9:40	0.74	0.55	14:30 − 14:40	0.54	0.73
9:40 − 9:50	0.71	0.73	14:40 − 14:50	0.60	0.75
9:50 − 10:00	0.73	0.77	14:50 − 15:00	0.71	0.77
10:00–10:10	0.69	0.6	15:00–15:10	0.77	0.73
10:10–10:20	0.57	0.54	15:10–15:20	0.58	0.72
10:20 − 10:30	0.53	0.73	15:20 − 15:30	0.63	0.68
10:30 − 10:40	0.62	0.61	15:30 − 15:40	0.64	0.86
10:40 − 10:50	0.63	0.71	15:40 − 15:50	0.63	0.68
10:50 − 11:00	0.54	0.87	15:50 − 16:00	0.54	0.66
11:00–11:10	0.57	0.68	16:00–16:10	0.66	0.57
11:10–11:20	0.81	0.70	16:10–16:20	0.67	0.67
11:20 − 11:30	0.70	0.63	16:20 − 16:30	0.66	0.66
11:30 − 11:40	0.63	0.67	16:30 − 16:40	0.66	0.71
11:40 − 11:50	0.50	0.63	16:40 − 16:50	0.68	0.78
11:50 − 12:00	0.36	0.24	16:50 − 17:00	0.70	0.71
			17:00–17:10	0.64	0.71
			17:10–17:20	0.59	0.60
12:00–14:00	Lunch break		17:20 − 17:30	0.73	0.73
			17:30 − 17:40	0.60	0.64
			17:40 − 17:50	0.52	0.47
			17:50 − 18:00	0.49	0.69
			9:00–18:00 average	0.62	0.66

## Conclusions

This study proposed a novel quantitative method to measure the productivity of office workers. The number of effective computer interactions per unit of time was used as a quantitative indicator to measure one’s work productivity in a non-invasive manner. During the 6-week test period, the study logged the effective computer interactions from 8 participants through in-house developed software and recorded the use of the sit-stand desk with displacement sensors. With a resolution of 10 min, we scrutinised the association between changes in productivity when participants took standing and sitting postures at work during the day. The main findings are as the followings:

- On average, each person changed to a standing posture about once a day for about 47 min each time;
- The changes to standing position occurred mainly between 2:30 pm and 4:00 pm, followed by the period between 10:30 am and 11:30 am;
- The productivity was higher in a standing posture than in a sitting posture during the day;
- When the test participants were allowed to freely adjust their postures, there was an immediate effect on the work productivity, with their productivity being approximately 6.5% higher than when they could only work in a sitting posture;
- Using a sit-stand desk had a noticeable effect on relieving musculoskeletal discomfort, especially in back pain and neck pain, while the effect on the knee and ankle varied from person to person.

While the findings of this experimental study suggest an immediate positive relationship between posture changes and productivity, we recognize that other unmeasured factors might also influence the results. Future extensions of this study could benefit from larger sample sizes and stratifying participants based on attributes such as gender, age, and other relevant criteria. Additionally, integrating comprehensive assessments of musculoskeletal health both before and after the study period would offer a more holistic understanding of the effects of posture changes.

## Data Availability

The datasets used and/or analysed during the current study are available from the corresponding author on reasonable request.
